# Development of a Sensitive Monoclonal Antibody-Based Colloidal Gold Immunochromatographic Strip for Lomefloxacin Detection in Meat Products

**DOI:** 10.3390/foods13162550

**Published:** 2024-08-16

**Authors:** Xinghua Zhou, Wenwen Pan, Na Li, Mahmoud Salah, Shuoning Guan, Xiaolan Li, Yun Wang

**Affiliations:** 1 School of Food and Biological Engineering, Jiangsu University, Zhenjiang 212013, China; zxh2012@ujs.edu.cn (X.Z.); 2222218062@stmail.ujs.edu.cn (W.P.); 15751011382@163.com (N.L.); 2222118132@stmail.ujs.edu.cn (S.G.); 2222318040@stmail.ujs.edu.cn (X.L.); 2 Department of Environmental Agricultural Science, Faculty of Graduate Studies and Environmental Research, Ain Shams University, Cairo 11566, Egypt; mahmoud.s.mostafa@hotmail.com

**Keywords:** lomefloxacin, colloidal gold immunochromatographic strip, ic-ELISA, monoclonal antibody

## Abstract

Lomefloxacin (LOM), an antibiotic crucial for preventing various animal diseases in animal husbandry, can pose serious health risks when found in excessive amounts in meat products. The development of highly specific and sensitive colloidal gold immunochromatographic test strips is essential for the accurate detection of this class of antibiotics. Our study utilized a monoclonal antibody (mAb) assay and immunochromatographic strips to detect lomefloxacin residues in meat products. The results showed minimal cross-reactivity with other structural analogs, with a maximum half inhibitory concentration (IC_50_) of 0.93 ng/mL and a linear range of 0.38 to 2.3 ng/mL for the indirect competitive enzyme-linked immunosorbent assay (ic-ELISA). The recovery of LOM was 80% to 120%, with an average coefficient of variation below 5%. The immunochromatographic strip test results showed a visual detection limit of 2.5 ng/g, meeting the market requirements for the test. This study highlights the significance of specific and sensitive testing methods for detecting lomefloxacin, ensuring consumers’ safety and health.

## 1. Introduction

Lomefloxacin (LOM) is a potent broad-spectrum fluoroquinolone (FQs) drug used to treat intestinal, urinary, and respiratory tract infections [1]. LOM can significantly reduce bacterial DNA helicase activity, inhibit bacterial growth, and achieve a powerful antibacterial effect, making it widely used in animal husbandry due to its high safety, efficiency, and wide antibacterial spectrum [2]. The use of LOM in animal agriculture raises food safety concerns due to residues in animal food leading to increased microbial resistance. Even small amounts of LOM in animal food can accumulate in the human body, causing direct harm and hindering growth and development, inducing bacterial resistance, and hinders the application of FQs in human antimicrobial therapy [3,4]. The problem of veterinary drug residues in animal food has become a global focus. The United States, Japan, the European Union, and China have regulations on drug residue limits [5]. In 2015, China’s Ministry of Agriculture banned the use of LOM in food animals and set new requirements for detecting LOM residues in animal foods [6]. Therefore, it is vital to develop a cost-effective test for detecting LOM residues in meat products.

Currently, detection methods for LOM residues in animal foods fall into two main categories. The first involves instrumental methods like High-Performance Liquid Chromatography (HPLC) [7], Spectrophotography [8], HPLC-MS/MS [9], capillary electrophoresis (CE) [10], and other methods. Such methods offer high accuracy and sensitivity but require specialized laboratories and long detection times [11]. The second category includes immunological rapid detection methods using antigen–antibody-specific binding, such as the enzyme-linked immunosorbent assay (ELISA) [12], colloidal gold immunochromatography (GICA) [13], and immunofluorescence technique [14], which are characterized by fast detection, easy operation, and high cost-effectiveness. Among them, ELISA and GICA are the most widely used rapid tests and have been commonly used for the on-site monitoring of drug residues in food. In terms of immunoassays for the detection of LOM, several research teams have developed ic-ELISA and colloidal gold immunochromatographic strips for the identification of LOM in milk. ic-ELISA has a limit of detection of up to 0.04 ng/mL [15]. The developed immunochromatographic strip device has a visual threshold of 5.0 ng/mL for the detection of LOM in labeled milk samples and has been shown to be cross-reactive with other quinolones [12]. However, the swift identification of LOM in meat products is seldom documented, which presents a significant challenge in meeting the market demand for rapid on-site detection of contaminated animal-derived foods.

In summary, the current research on colloidal gold immunochromatographic test strips for LOM is limited. Achieving the specific detection of LOM has been a challenge. Therefore, the development of a highly specific and sensitive colloidal gold immunochromatographic strip for detecting this class of antibiotics holds great significance. In this study, we have prepared a monoclonal antibody (mAb) with a high sensitivity and affinity for LOM. Additionally, mAb-based ic-ELISA and colloidal gold immunochromatographic strips have been established to detect LOM rapidly.

## 2. Materials and Methods

### 2.1. Reagents and Equipment

LOM, norfloxacin (NOR), pefloxacin (PEF), ofloxacin (OFL), bovine serum albumin (BSA), ovalbumin (OVA), and other reagents used for the preparation of antigens and immunizations were purchased from Sigma-Aldrich (St. Louis, MO, USA). All cell culture reagents were purchased from Thermo Fisher Scientific (Shanghai, China). Antibody subtype identification kits were purchased from Luoyang Biotone Experimental Materials Center (Luoyang, China). All other reagents and chemicals were purchased from Sinopharm Chemical Reagent Co. (Shanghai, China). Nitrocellulose (NC) membranes, glass fiber membranes, absorbent pads, polyvinyl chloride sheets, and filter paper were purchased from Kinbio Tech Co. (Shanghai, China).

A UV–vis spectrometer (GENESYS) and LC-MS/MS were obtained from Thermo Fisher Scientific (Waltham, MA, USA). Enzyme-labeling apparatus (1201-7044) was purchased from Thermo Fisher Scientific (Shanghai, China). The XYZ 3D Film Scribing and Gold Spraying Instrument (HM 3035) were by Gold Standard Biotechnology Co. Ltd. (Shanghai, China). 

Female BALB/c mice (SPF grade) aged 6–8 weeks were purchased from Changzhou Cavins Laboratory Animal Co. Ltd. (Changzhou, China) (License No.: SCXK (Su) 2016-0010) and kept and subsequently immunized at the Animal Experiment Center of Jiangsu University (License No.: SYXK (Su) 2018-0053). All animal experiments were in accordance with the Guidelines for the Management and Use of Laboratory Animal Breeding, and humanitarian principles were followed for laboratory animals.

### 2.2. Synthesis and Identification of Complete Antigens

**Figure 1:**
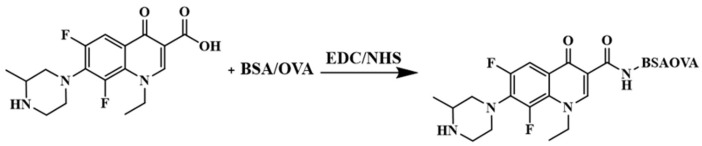
Synthesis of the immunogen antigen and coating antigen of LOM.

LOM is directly conjugated with BSA and OVA to form the desired immunogen and envelope by an activated ester method [16], respectively (Figure 1). Attention to detail is crucial in the preparation of the protein–hapten conjugate. In a meticulous process, carefully mix 3.2 mg of LOM, 2 mg of N-hydroxysuccinimide (NHS), and 3.5 mg of 1-ethyl-3-(dimethylaminopropyl) carbodiimide hydrochloride (EDC) in 0.3 mL of dimethylformamide (DMF). Allow the solution to activate overnight at room temperature. Next, add the activated hapten solution drop by drop to 5 mL of protein solution, suspended in a carbonate (CBS) buffer containing either BSA (11 mg/mL) or OVA (8 mg/mL). The ensuing reaction should be stirred overnight at room temperature. Subsequently, after the reaction, dialyze the solution using a dialysis cut-off of 8000–14,000 Dalton (DA) in phosphate buffer at pH 7.4 for 72 h at 4 °C. It is imperative to replace the dialysate three times a day. Post dialysis, store the solution at −20 °C for future use. These carefully executed steps ensure the production of a high-quality protein–hapten conjugate. Finally, UV–vis spectroscopy [17,18] is utilized to confirm whether the LOM is successfully coupled to the carrier protein. 

### 2.3. Preparation and Identification of mAb against LOM

#### 2.3.1. Mouse Immunization

The mAb was prepared by mouse immunization and the hybridoma technique. The prepared immunogen was diluted to 2 mg/mL in saline, and an equal volume of Fuchs’ Complete Adjuvant (FCA) was added and fully emulsified using an emulsifier. The mice were immunized with 100 µg of FCA in multiple subcutaneous injections into the back of the body for the first immunization (primary immunization). The mice were then boosted every three weeks with a 2 mg/mL dilution of the immunogen and an equal volume of Fuchs’ Incomplete Adjuvant (FIA), fully emulsified in the same manner as the first immunization, with a volume of 50 µg per mouse, and from the third immunization onwards, the tail serum of the mice was tested in an ic-ELISA, to select the mice that have a high potency and inhibitory rates for cell fusion. 

#### 2.3.2. Cell Fusion

SP2/0 myeloma cells were fused with splenocytes into 1 mL of PEG solution at a ratio of 7:1 [19,20], and then, the fused cells were cultured in HAT medium, and half-exchange and full-exchange cultures were carried out on the third and sixth days after cell fusion, respectively, and on the seventh day, the positive wells were screened with the ic-ELISA method, and subcloned three times with the limiting dilution method. The hybridoma cells with the best affinity and inhibitory effect were injected intraperitoneally into mice to produce mAb, and the ascites were collected and purified by ammonium sulfate precipitation.

#### 2.3.3. Identification of mAb against LOM

The isotype of mAb was determined by the isotype kit, and the affinity constant (*Ka*) was determined with the coated antigen and mAb dilution method by ELISA [21].

The affinity constant (*Ka*) was calculated according to the following formula [21]:

$$Ka=\frac{n-1}{2(n\left[Ab\right]t-\left[Ab^{\prime }\right]t)}$$

Note: $n=\left[Ag\right]t/\left[Ag^{\prime }\right]t$, where $\left[Ag\right]t$ and $\left[Ag^{\prime }\right]t$ represent two different encapsulation concentrations, and $\left[Ab\right]t$ and $\left[Ab^{\prime }\right]t$ represent the concentration of LOM-mAb at 50% OD_450nm_ at these two encapsulation concentrations. 

### 2.4. Optimization of ic-ELISA

There are many factors affecting the sensitivity of the ic-ELISA method, and the optimization experiments are generally based on the conditions of plate wrapping, closure conditions, color development time, and standard diluent [22,23,24,25,26,27]. In this study, we determined the optimal encapsulation concentration and the concentration of added antibody by the checkerboard method and then optimized the containment conditions, competition time, and standard diluent (NaCl content and pH value in the buffer) using the ratio of the maximum absorbance value (A_max_) and the IC_50_ as criteria [22,23,24,25,26,27].

### 2.5. Identification of mAb Sensitivity and Specificity

The sensitivity of the mAb was evaluated by half-maximum inhibitory concentration measurement (IC_50_) [28]. Firstly, the optimal concentrations of LOM-OVA and anti-LOM mAb were obtained by the checkerboard method. Then, indirect competitive inhibition curves (with the OD_450_ value as the vertical coordinate and the logarithm of the LOM concentration as the horizontal coordinate) were established by ic-ELISA and Origin 2021 software, and the IC_50_ was calculated by this method.

Specificity was expressed as the crossover rate (*CR*), and the IC_50_ of the related structural analogs was determined by ic-ELISA. The crossover rate was calculated according to the following formula:

$$CR(\%)=(\frac{{IC}_{50}\;of\;LOM}{{IC}_{50}\;of\;analogues})\times 100\%$$

### 2.6. Preparation of Colloidal Gold-Labeled mAb

Colloidal gold was prepared by the classical trisodium citrate reduction method [29,30]. After improvement, the following specific method was finally obtained: 100 mL of 1% chloroauric acid was taken in a flask, heated to boiling, and then 4 mL of 1% aqueous trisodium citrate solution was added under stirring conditions. Continue heating and boiling; in the process, the color of the solution will change until the solution shows red; stop heating, and stop stirring when the temperature drops to room temperature, at which time, the solution should be sealed and stored at 4 °C for use. The colloidal gold was characterized by UV spectral scanning and transmission electron microscopy (TEM) scanning.

Take 3 mL colloidal gold solution and adjust the pH to 8.5 with 0.1M K_2_CO_3_ solution. Then, 6 μL antibody solution (3 μg/μL) is slowly added, and the reaction is carried out at 20–25 °C for 50 min. Then, 300 μL 10% (*w*/*v*) BSA solution is slowly added to the above solution and incubated at room temperature for 30 min to block the non-specific binding site, and then centrifuged at 8000 rpm for 15 min. Finally, the precipitated colloidal gold-labeled monoclonal antibody was resuspended in PBS (0.01 M pH 7.4) containing 2% (*w*/*v*) BSA, 1% (*w*/*v*) sucrose, 0.5% Tweene-20, and 0.02% (*w*/*v*) sodium azide and stored at 4 °C in the dark. 

### 2.7. Preparation of Colloidal Gold Immunochromatographic Strip

**Figure 2:**
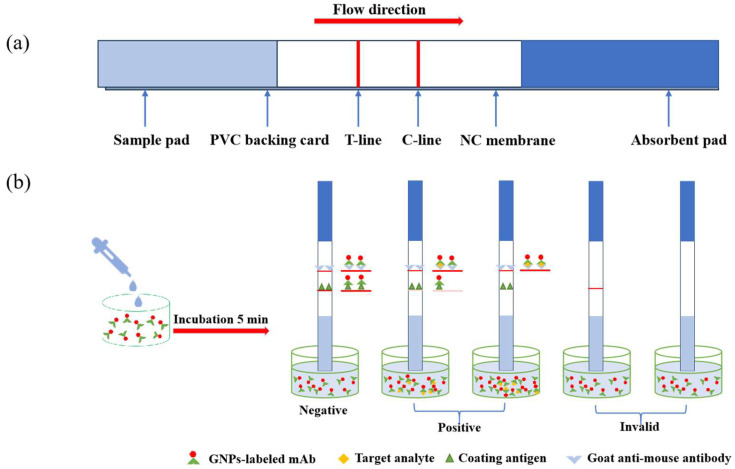
Illustration of colloidal gold immunochromatographic strip: (**a**) Structure and (**b**) principle.

The colloidal gold immunochromatography strip was developed as previously reported [31]. The colloidal gold immunochromatography strip is mainly composed of a sample pad, bottom plate (PVC), nitrocellulose film (NC film), and absorbent pad, as shown in Figure 2a. The test line (T-line) and the quality control line (C-line) constitute the detection area, wherein the T line is formed by the coating and fixed on the NC film, and the C line is formed by the sheep anti-mouse antibody (second antibody) and fixed on the NC film. The PVC base plate at the bottom supports the other parts, with the NC membrane in the middle, and the sample pads and absorbent pads on both sides, which overlap the NC membrane by 1–2 mm. In addition, the sample pads should be soaked in the treatment solution for 5 h to reduce the matrix interference before assembling, and then placed in the oven at 37 °C for drying. After the assembly of the test strips, they were processed into 3–4 mm strips for use. The prepared strips were sealed and stored under sufficiently dry conditions.

### 2.8. Principle and Use of Immunochromatographic Test Strips

The basic principle is shown in Figure 2b. The strip is inserted into the microhole, and under the action of the capillary tube, the liquid in the microhole will move spontaneously from the sample pad to the direction of the suction end. If the sample is negative, the gold-labeled antibody in the mixture will bind to the T-line coating as much as possible, and the excess gold-labeled antibody will continue to move forward and bind to the secondary antibody of the C-line; so if the result is negative, two red bands of similar colors will appear on the test strip or the T-line will be slightly darker than the C-line. If the result is positive, a large number of gold-labeled antibodies will bind to the test substance and cannot bind to the coated substance, resulting in a lighter T-line, until it disappears (usually the minimum concentration of the test substance that begins to become a lighter T-line is set as the vLOD of the test strip); so if the result is positive, the color of the T-line must be lighter than that of the C-line. In addition, because the C-line is fixed with sheep anti-mouse antibody, no matter whether the test sample is positive or not, the sheep anti-mouse antibody will react specifically with the gold-labeled antibody. Therefore, if the C-line disappears during the test, it means that the test strip is unqualified.

The specific experimental steps of the immunochromatographic test strips were as follows: 150 μL of sample extract or 150 μL of standard was taken into the microtiter wells of the enzyme plate, and then 50 μL of diluted gold standard antibody solution was added into each well and mixed well and then incubated for 5 min under the condition of 20–25 °C, and then the prepared strips were inserted into the microtiter wells, and then the color development of the T-line and the C-line was observed after 10 min.

### 2.9. Optimization of Strip Test Conditions

#### 2.9.1. Selection of the pH of the Labeled Antibody

The pH of the best labeling antibody in the test was adjusted by 0.1 M K_2_CO_3_. Take 7 centrifuge tubes, add 1mL of colloidal gold solution, then add 0, 4, 6, 8, 10, 12, 14, 16, and 20 μL of 0.1 M K_2_CO_3_ solution. After mixing well, add 10 μL of 0.5 mg/mL prepared monoclonal antibody, mix well, and leave for 30 min; observe the color change of the solution. The group with no change in the color of the colloidal gold solution and the smallest volume of K_2_CO_3_ solution was selected.

#### 2.9.2. Selection of Labeled Antibody Volume

Select the amount of K_2_CO_3_ solution optimized in the above test, add 1 mL of colloidal gold solution to 8 centrifuge tubes, and then add 4, 6, 8, 10, 12, 14, 16, and 20 µg of antibody in sequence, and observe the color change after 5 min of standing at room temperature. Under the condition that the color remains unchanged, the group with the lowest amount of antibody added is selected.

#### 2.9.3. Selection of T-Line Coating Concentration

Spray 0.2, 0.5, and 1 mg/mL of the encapsulant on the T-line, dry it, and then carry out the color development reaction, and select the group with the best color development as the optimal concentration of the encapsulant.

### 2.10. Testing of Actual Samples

In this study, pork, fish, and chicken were selected for the detection of actual samples. First, pork, chicken, and fish that had been identified as negative by LC-MS/MS were used for the labeling recovery verification of these three types of substances. The nitrogen-blowing method used here was used for sample pretreatment, and the specific experimental operation referred to Announcement No. 1025 of the Ministry of Agriculture 8-2008 (Detection of fluoroquinolone residues in animal food—enzyme-linked immunosorbent assay). In addition, 0, 2, 2.5, and 3 ng/g were selected as the additive concentrations of LOM for detection. The vLOD of the test strip was obtained by visual observation.

## 3. Results and Discussion

### 3.1. Identification of LOM Complete Antigen

LOM is a small molecule (relative molecular weight <500 Da) that is only immunoreactive but not immunogenic, and cannot directly stimulate the body to produce an immune response and thus produce antibodies. Only by coupling with macromolecular carrier proteins to form complete antigens can it stimulate the body to produce an immune response and thus produce antibodies [32,33]. Small molecules with amino or carboxyl groups can be coupled to carrier proteins to form complete antigens, and small molecules without amino or carboxyl groups need to introduce new active groups to be coupled to carrier proteins. Due to the presence of carboxyl group at the C-3 position of fluoroquinolone antibiotics, LOM can be directly ligated to carrier proteins to synthesize complete antigens by the activated ester method.

**Figure 3:**
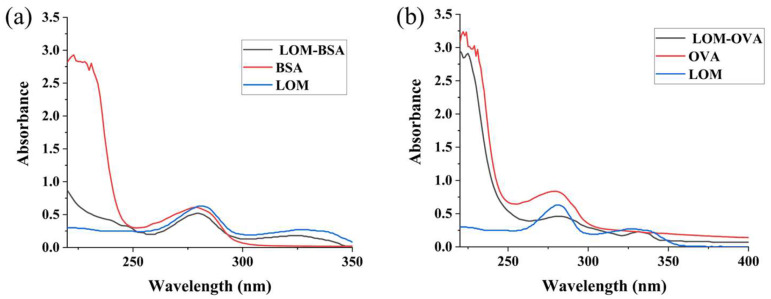
UV–vis spectrum of LOM immunogen (**a**) and coating antigen (**b**).

Figure 3 shows the UV spectral scan of immunogenic LOM-BSA and the coated antigen LOM-OVA. From Figure 3a, it was found that the maximum characteristic absorption peak of immunogenic LOM-BSA was shifted relative to that of LOM and BSA, and the peak shape changed to a certain extent, which indicated that the immunogenic synthesis of LOM had been successful [34]. Similarly, the original LOM-OVA was also successfully synthesized.

### 3.2. Screening of Hybridoma Cells

Three BALB/c mice were injected five times with the prepared immunogen, and the antiserum was determined by ic-ELISA after the fifth immunization. The results are shown in Table 1, in which the concentration of the coated stock solution used was 0.3 μg/mL and the antiserum was diluted 3000 times. Among the three mice immunized with LEM-BSA, the inhibition rate of mouse No. 3 was better than that of mouse No. 2, but the titer was significantly lower, which would greatly reduce the probability of successful cell fusion; meanwhile, titer No. 2 was not much different from titer No. 1, but its inhibition rate was significantly lower, so mouse No. 1 was selected for subsequent cell fusion experiments.

**Figure 4:**
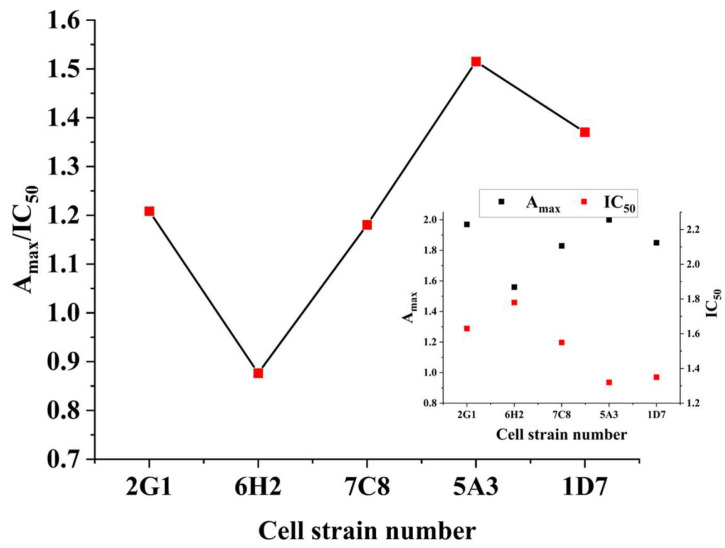
Screening results of hybridoma cell of LOM.

The inhibition was detected by ELISA during the three subclones, and the IC_50_ of the five cell lines with good effect was detected at the third subclone. Figure 4 shows the cell screening of the third subclone of LOM hybridoma cells. It can be seen from the figure that although the titers of cell line 7C8 and cell line 1D7 are close, the IC_50_ and Amax/IC_50_ values of 1D7 are better than those of 7C8. Although the IC_50_ of cell line 1D7 was similar to that of 5A3, the Amax and Amax/IC_50_ values of 5A3 were both greater than 1D7. Therefore, 5A3 was selected as the best hybridoma cell line for the subsequent preparation of a LOM monoclonal antibody to establish related detection methods.

### 3.3. Identification of mAb

**Figure 5:**
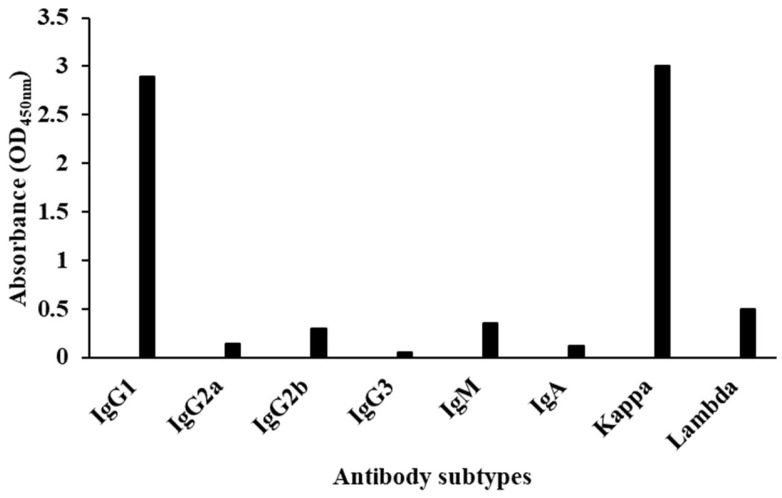
The results of antibody isotypes.

**Figure 6:**
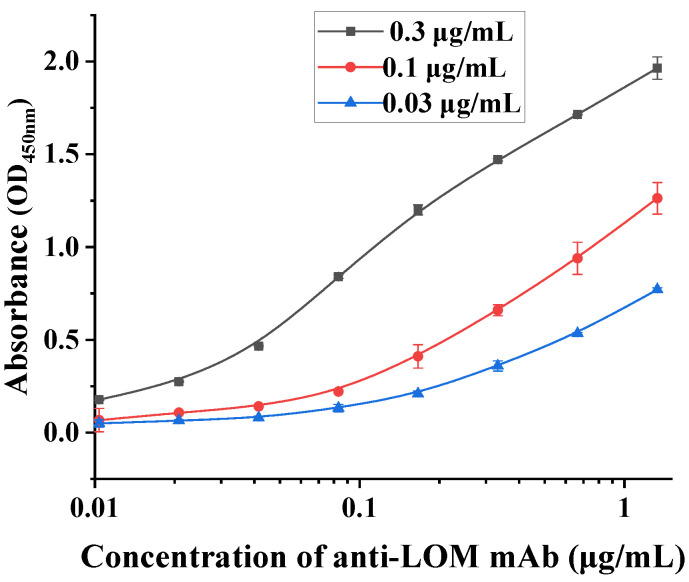
Affinity constant result of LOM antibody.

The isoforms of the prepared monoclonal antibodies were characterized by commercial kits. The results are shown in Figure 5. The isoform of the LOM antibody was IgG1, and the light chain was Kappa. Three coated antigen concentrations, 0.3, 0.1, and 0.03 µg/mL, were chosen to determine the affinity constant of the mAb. The results in Figure 6 showed that the affinity constant of the mAb was 1.52 × 10^10^ L/mol, indicating that the LOM antibody prepared in this study had a high affinity and can be used to establish relevant immunological assays.

### 3.4. Establishment and Optimization of ic-ELISA

According to the checkerboard method, the optimal concentrations of LOM coated antigen and antibody were 0.03 μg/mL and 0.19 μg/mL, respectively, which were optimized at this working point.

**Figure 7:**
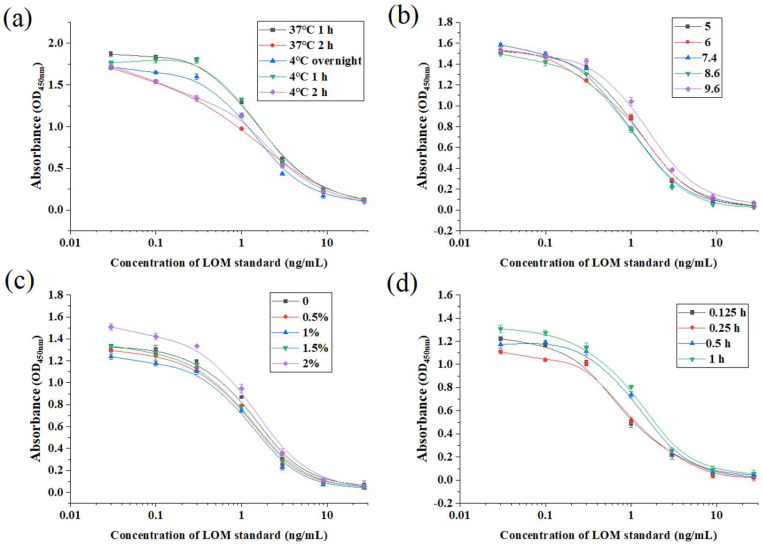
Effect of different blocking conditions (**a**), competition time (**b**), NaCl content (**c**), and pH (**d**) on the LOM ic-ELISA method.

According to Figure 7a and Table 2, the Amax is larger than the other two types when the sealing condition is incubated at 37 °C for 1 h and 4 °C for 1 h and 2 h, which may be caused by the non-specific adsorption of part of the antibodies or the binding of antibodies and detection substances to the bottom of the plate due to insufficient sealing. However, the IC_50_ value of 37 °C incubated for 2 h is lower than that of 4 °C incubated for 2 h, and the Amax/ IC_50_ value of 37 °C incubated for 2 h is significantly higher than that of 4 °C incubated for 2 h, so 37 °C incubated for 2 h is the optimal sealing condition.

It can be seen from Figure 7b and Table 2 that both the Amax and IC_50_ gradually increase with the extension of competition time before 0.5 h, and both reach equilibrium when they reach 0.5 h, and there is little difference between 0.5 h and 1 h. Therefore, this study sets 0.5 h (30 min) as the optimal competition time.

As can be seen from Figure 7c and Table 3, the IC_50_ and Amax/IC_50_ are better than the other groups when the NaCl content is 1.5%, but there is no obvious advantage. Here, PBS with 1.5% NaCl content is selected to dilute the standard product.

As can be seen from Figure 7d and Table 3, the Amax, IC_50_ and Amax/ IC_50_ are the best when the pH is 7.4; that is, the sensitivity is the best when the working environment is neutral. Therefore, 7.4 is selected as the optimal pH of the working environment.

In summary, the optimal sealing condition of the ic-ELISA method for LOM is 37 °C for 2 h, the optimal competition time is 0.5 h, the optimal NaCl content of the standard diluent is 1.5%, and the optimal pH is 7.4.

### 3.5. Specificity of mAb

Table 4 shows the results of the crossover rate assays for LOM and its five major structural analogs. As can be seen from the table, the crossover rates of the LOM monoclonal antibodies prepared in this paper with PEF, OFL, MBF, CIP, and NOR are all lower than 0.1%, indicating that the monoclonal antibodies prepared in this paper have a high specificity. The cross-reactivity rate between LOM mAb and NOR prepared by Cao [35] et al. was 17.5%. The LOM monoclonal antibody prepared by Mukunzi [28] et al. had a crossover rate of 111.8% with NOR, 28.81% with CIP, and 28.23% with OFL. Compared with previous studies, the LOM monoclonal antibody prepared in this study has obvious specific advantages. It provides a guarantee for more the sensitive and rapid detection of LOM, a prohibited veterinary drug.

### 3.6. Preparation of Colloidal Gold-Labeled mAb

**Figure 8:**
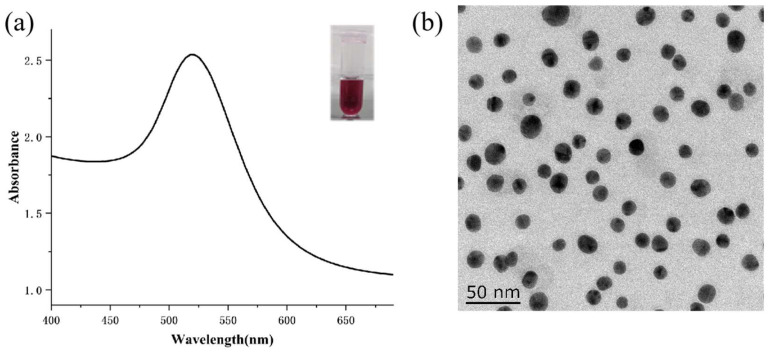
The characterization of colloidal gold: (**a**) UV-vis spectrum and (**b**) TEM image.

It can be seen from Figure 8a that the colloidal gold nanoparticle solution prepared in this study is wine-red in color, clear and transparent without impurities. At the same time, it can be seen from the ultraviolet scanning image that the colloidal gold nanoparticle prepared has a maximum light absorption value at 522 nm, and the peak width is relatively narrow, which is similar to the results reported by Wang Zhongxing et al [36]. This indicates that colloidal gold nanoparticles have been successfully prepared in this study.

The quality of the colloidal gold is a key factor affecting the sensitivity and stability of the test strip [37]. Colloidal gold with a particle size between 15 and 40 nm is often used in the preparation of immunochromatographic test strips. On the one hand, if the particle size of the colloidal gold is too small, it cannot form an obvious strip on the test strip. On the other hand, if the particle size is too large, it is easy to cause the aggregation of colloidal gold nanoparticles, mainly because the dispersion of colloidal gold nanoparticles is not good. It can be seen from Figure 8b that the colloidal gold prepared in this study has a uniform particle size and good dispersion, with a particle size of about 16 nm.

**Figure 9:**
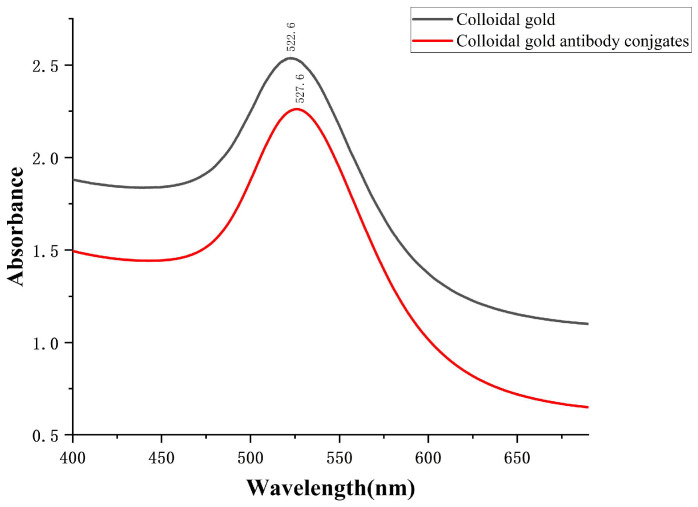
The UV–vis spectrum of colloidal gold and colloidal gold-labeled anti-LOM mAb.

Figure 9 depicts the UV-vis spectra of the colloidal gold and its conjugate. The maximum absorption wavelength for colloidal gold is 522 nm, whereas for colloidal gold-labeled anti-LOM mAb, it is 527 nm. This discrepancy is due to the broadening and red-shifting of the surface plasma band resulting from the interaction between the antibody and the colloidal gold particles [37].

### 3.7. Determination of the Parameters for the Strip Test Optimization

Under weak base conditions, colloidal gold is more inclined to binding with antibodies. In this experiment, K_2_CO_3_ solution was selected to adjust the pH, and the color change of gold-labeled antibodies under different antibody concentrations was observed by the naked eye. If the number of antibodies added was too low—that is, the added antibodies could not keep the colloidal gold stable—the solution would produce a precipitation phenomenon from red to blue. The color of the solution remains the same if the minimum number of antibodies is reached or exceeded, to stabilize the colloidal gold. Generally, the minimum amount of antibody that keeps the color of the colloidal gold solution unchanged is used as the minimum amount of labeled antibody [36], but in practice, due to the influence of factors such as antibody purity and synthesis efficiency, the amount of labeled antibody obtained in the experiment is generally increased by 10–20% [36]. Therefore, based on the minimum amount, by increasing the amount of monoclonal antibody by 15%, the following conclusions can be drawn from Table 5 and Table 6: the best conditions for the macroscopic determination of LOM colloidal gold strips are 1mL colloidal gold, 8 μL K_2_CO_3_ solution, and 6.9 μg antibody.

Under the conditions of an optimal pH and amount of labeled antibody, the coating reserve solution was diluted into different concentrations and sprayed on NC film for a color development reaction. The optimal concentration of coating solution for the colorimetric detection of LOM colloidal gold test paper is 0.5 mg/mL.

### 3.8. Ic-ELISA and the Colloidal Gold Immunochromatographic Strip for LOM Detection

As can be seen from Table 7, the recoveries of added LOM for pork, grass carp, and chicken are 91.0%~104.2%, 86.1%~110.4%, and 83.0%~96.4%, respectively, all within the reasonable range of 80%~120%, with an average coefficient of variation less than 5%. This shows that the method established in this paper has a good accuracy in these three types of meat products, indicating that the method established in this paper is feasible for the actual detection of LOM residues in these three types of meat products.

**Figure 10:**
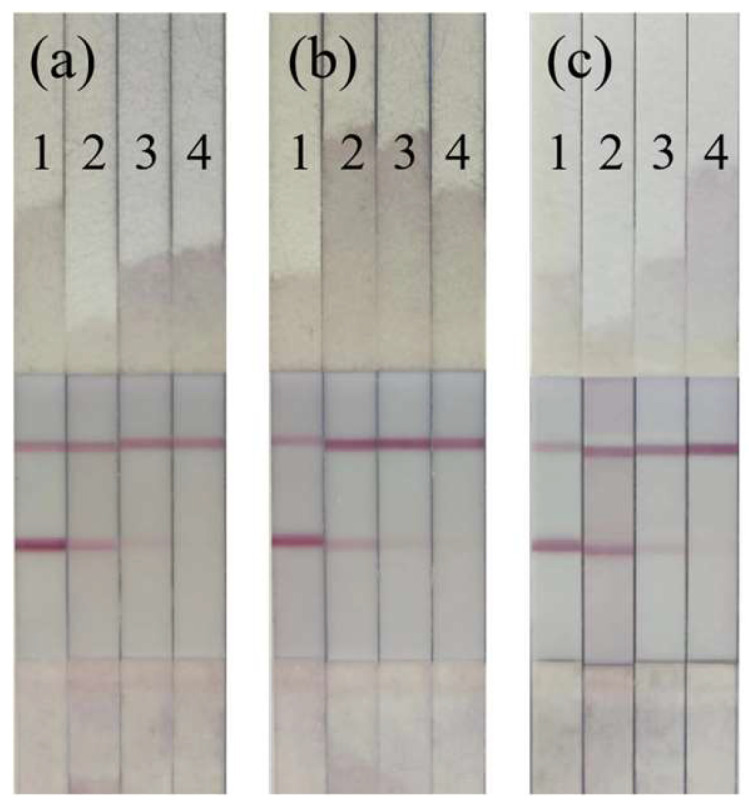
Images of colloidal gold immunochromatographic strip tests for LOM in pork, fish and chicken samples. Note: The added concentrations are 0, 2, 2.5, and 3 ng/g from left to right, where (**a**) is the detection in pork samples, (**b**) is the detection in fish samples, and (**c**) is the detection in chicken samples.

Compared with the negative group, the detection limit of the test strip is defined as the lowest concentration when the color of the T-line is lighter than that of the C-line [38]. Figure 10 shows the detection of LOM residues in pork, fish, and chicken by colloidal gold immunochromatographic test strips. It can be seen from the figure that the labeling detection of the three kinds of meat is the same, which indicates that the test strips prepared in this study have good stability. When the additive concentration is 0—that is, negative—the strip color of the T-line is the same as that of the C-line or darker, and the color of the T-line gradually becomes lighter as the additive concentration increases, and the detection conditions of the three types of meat are similar. In the actual samples, the vLOD is 2.5 ng/g. According to the statistics, the vLOD of the test strips used to detect LOM can reach 5 ng/mL at present, but it is mainly for the detection of a broad spectrum [39], which is not conducive to the detection of such prohibited antibiotics as LOM in animal food, and there is less specific detection. Therefore, the colloidal gold immunochromatographic test strip developed in this paper provides a strong guarantee for the detection of LOM. Table 8 (comparison with other measurement methods) is a comparison of the detection limit and detection time with other methods, and it provides more evidence that our established method is sensitive, fast, and simple.

## 4. Conclusions

In this study, the mAb to LOM was obtained by screening 5A3 hybridoma to prepare ascites, and its affinity constant was 1.52 × 10^10^ L/mol. A highly sensitive monoclonal antibody-based ic-ELISA and gold immunochromatographic strips were developed for the detection of LOM in meat products. The recovery rates of pork, grass carp, and chicken were 91.0%~104.2%, 86.1%~110.4%, and 83.0%~96.4%, respectively, with an average CV less than 5%. The vLOD of pork, grass carp, and chicken samples in colloidal gold immunochromatographic strips was 2.5 ng/g. Compared with other methods, this method is more sensitive, rapid, and suitable for the on-site detection of LOM, and it can provide a technical reference for the rapid detection of pesticide and veterinary drug residues, as well as other hazardous factors in food. Although the immunochromatographic test strip has the advantage of rapid detection, later studies can combine it with traditional laboratory detection methods to improve the accuracy and reliability of detection, to improve the level of food safety, and to protect people’s life and health.

## Figures and Tables

**Table 1:** Results of five immune serum determinations in mice immunized with three immunogens.

Immunogen	LOM-BSA		
Mouse Number	1	2	3
Titer (OD_450nm_)	2.135	1.995	1.201
Inhibition ratio (%)	61	32	62

Note: LOM plus scalar is 25 ng/mL.

**Table 2:** Optimization of blocking conditions and competition time on LOM ic-ELISA.

	Blocking Conditions					Competition Time/h			
	37 °C 1 h	37 °C 2 h	4 °C Overnight	4 °C 1 h	4 °C 2 h	0.125	0.25	0.5	1
A_max_	1.89	1.756	1.781	1.813	1.799	1.319	1.35	1.406	1.453
IC_50_	1.65	1.16	1.38	1.72	1.63	0.73	0.92	1.14	1.15
A_max_/IC_50_	1.15	1.51	1.29	1.05	1.1	1.81	1.47	1.23	1.26

**Table 3:** Optimization of NaCl content and pH on LOM ic-ELISA.

	NaCl Content					pH				
	0%	0.5%	1%	1.5%	2%	5	6	7.4	8.6	9.6
A_max_	1.45	1.43	1.42	1.44	1.46	1.56	1.57	1.62	1.61	1.62
IC_50_	1.28	1.15	1.14	1.12	1.34	1.16	1.12	0.93	0.98	1.45
A_max_/IC_50_	1.13	1.24	1.25	1.29	1.09	1.34	1.4	1.74	1.64	1.12

**Table 4:** The cross-reactivity results of LOM monoclonal antibody.

Compound	IC_50_ (ng/mL)	CR (%)
LOM	0.93	100
PEF	>500	<0.1
OFL	>500	<0.1
MBF	>500	<0.1
CIP	>500	<0.1
NOR	>500	<0.1

**Table 5:** The color of antibody-labeled gold nanoparticles under different pH antibody concentrations.

	1 mL Colloidal Gold	Optimization							
pH	K_2_CO_3_ (μL)	0	4	6	8	12	14	16	20
	Color	−−+	−−+	−++	+++	+++	+++	+++	+++
Antibody	LOM antibody (μg)	4	6	8	10	12	14	16	20
	Color	−−+	+++	+++	+++	+++	+++	+++	+++

Note: −−+: bluish violet; −++: purple; +++: red.

**Table 6:** The optimization for the concentrations of antigen and antibody on the strip.

Antigen Concentration (mg/mL)	0.2			0.5			1		
Gold-labeled antibody (μL)	4	8	12	4	8	12	4	8	12
Negative T-line gray value	355	495	692	602	1192	1521	1253	1641	2015
vLOD (ng/mL)	-	1	1	1	2	2	2	5	10

Note: -: the T-line signal cannot be detected by the naked eye.

**Table 7:** Recovery rates of LOM (*n* = 3).

Samples	Spiked Level (ng/g)	Recovery (%)	CV (%)
Pork	0.00	-	-
	1.00	91.00 ± 1.00	6.12
	2.00	104.20 ± 2.20	3.51
Grass carp	0.00	-	-
	1.00	86.10 ± 2.00	4.22
	2.00	110.40 ± 2.60	4.58
Chicken	0.00	-	-
	1.00	83.00 ± 5.00	3.67
	2.00	96.40 ± 4.20	5.13

**Table 8:** Comparison of this method with other measurement methods.

Reference	LOD (ng/mL)	Method	Analysis Time	Detection Object
This work	0.38	ELISA	5 h	Pork, fish, and chicken
	2.5	Colloidal gold immunochromatographic strip	10 min	
6	20	HPLC	1–2 h	Seminal plasma samples
7	17.7	Spectro photography	1–2 h	Tablets
8	0.016–0.052	HPLC-MS/MS	1–2 h	Water and biological fluids
9	0.028–0.094	Capillary electrophoresis	5 h	Milli-Q, mineral, tap, and wastewater
10	0.19	ELISA	5 h	Bovine milk
	5	Colloidal gold immunochromatographic strip	15 min	
11	3.8	Spectrofluorimetric	1–2 h	Tablet formulations and in real human plasma

## Data Availability

The original contributions presented in the study are included in the article, further inquiries can be directed to the corresponding author.

## References

[1] Patel K., Goldman J.L. (2016). Safety Concerns Surrounding Quinolone Use in Children. J. Clin. Pharmacol..

[2] Zhang H.T., Jiang J.Q., Wang Z.L., Chang X.Y., Liu X.Y., Wang S.H., Zhao K., Chen J.S. (2011). Development of an indirect competitive ELISA for simultaneous detection of enrofloxacin and ciprofloxacin. J. Zhejiang Univ. Sci. B.

[3] Kumar N., Rosy, Goyal R.N. (2017). Gold-palladium nanoparticles aided electrochemically reduced graphene oxide sensor for the simultaneous estimation of lomefloxacin and amoxicillin. Sens. Actuators B Chem..

[4] Orachorn N., Bunkoed O. (2019). A nanocomposite fluorescent probe of polyaniline, graphene oxide and quantum dots incorporated into highly selective polymer for lomefloxacin detection. Talanta.

[5] Tang X.-Y., Zheng Z., Wang M., Guo L.-Y. (2017). Current status and development of standards for veterinary drug residue limits in livestock and poultry products. J. Food Sci. Technol..

[6] Du F.-B., Tan J.-H. (2007). Synthesis and identification of Lomefloxacin artificial antigen. J. Vet. Med..

[7] Carlucci G., Mazzeo P., Vetuschi C. (2003). Development and validation of an HPLC method for determination of lomefloxacin in seminal plasma involving solid-phase extraction (SPE). J. Liq. Chromatogr. Relat. Technol..

[8] El-Hamshary M.S., Hanafi R.S., Fouad M.A., Al-Easa H.S., El-Moghazy S.M. (2021). Spectrophotometric and spectrofluorimetric determinations of lomefloxacin lanthanum complex in tablets using multivariate modeling and optimization stratagem. J. Iran. Chem. Soc..

[9] Jian N.-G., Liang S.-H., Cao J.-K., Di Q.-N., Kang K., Xu Q. (2019). A nanofiber mat prepared from sulfonated polyaniline for solid-phase extraction of fluoroquinolones from water and biological fluids prior to their quantitation by UPLC-MS/MS. Microchim. Acta.

[10] Herrera-Herrera A.V., Ravelo-Perez L.M., Hernandez-Borges J., Afonso M.M., Antonio Palenzuela J., Angel Rodriguez-Delgado M. (2011). Oxidized multi-walled carbon nanotubes for the dispersive solid-phase extraction of quinolone antibiotics from water samples using capillary electrophoresis and large volume sample stacking with polarity switching. J. Chromatogr. A.

[11] He X.-T., Chen Z.-J., Huang S., Xiao Z.-M., Liu J., Zhong M., Wang H., Shen Y.-D., Xu Z.-L. (2023). Rapid detection of preservative in vegetables by colloidal gold immunochromatography based on nanoantibodies. Food Sci..

[12] Mukunzi D., Isanga J., Suryoprabowo S., Liu L., Kuang H. (2017). Rapid and sensitive immunoassays for the detection of lomefloxacin and related drug residues in bovine milk samples. Food Agric. Immunol..

[13] Wu Y.X., Wu M.J., Liu C., Tian Y.C., Fang S.Q., Yang H., Li B., Liu Q. (2021). Colloidal gold immunochromatographic test strips for broad-spectrum detection of Salmonella. Food Control.

[14] Kudose S., Sekulic M., Walavalkar V., Batal I., Markowitz G.S., D’Agati V.D., Santoriello D. (2023). Immunofluorescence Staining for IgG Subclass: Cause for Discrepancy in the Detection of IgG1. Kidney Int. Rep..

[15] Cao Z., Lu S., Liu J., Zhan J., Meng M., Xi R. (2011). Preparation of Anti-Lomefloxacin Antibody and Development of an Indirect Competitive Enzyme-Linked Immunosorbent Assay for Detection of Lomefloxacin Residue in Milk. Anal. Lett..

[16] Lei X., Xu L., Song S., Liu L., Kuang H. (2018). Development of an ultrasensitive ic-ELISA and immunochromatographic strip assay for the simultaneous detection of florfenicol and thiamphenicol in eggs. Food Agric. Immunol..

[17] Zeng H., Chen J., Zhang C., Huang X.-a., Sun Y., Xu Z., Lei H. (2016). Broad-Specificity Chemiluminescence Enzyme Immunoassay for (Fluoro)quinolones: Hapten Design and Molecular Modeling Study of Antibody Recognition. Anal. Chem..

[18] Wu Y., Guo S., Dong Q., Song Y. (2016). Development of an Immunochromatographic Test Strip for Rapid Simultaneous Detection of Enrofloxacin and Ofloxacin in Tissue of Chicken Muscle and Pork. Food Anal. Methods.

[19] Li J., Zhi A., Jia G., Nie H., Ai L. (2018). Development of an ic-ELISA and immunochromatographic strip for detection of sparfloxacin in honey. Food Agric. Immunol..

[20] Liu B.-H., Hung C.-T., Lu C.-C., Chou H.-N., Yu F.-Y. (2014). Production of Monoclonal Antibody for Okadaic Acid and Its Utilization in an Ultrasensitive Enzyme-Linked Immunosorbent Assay and One-Step Immunochromatographic Strip. J. Agric. Food Chem..

[21] Wang B., Xiao K., Meng L., Shou C. (2020). Generation and characterization of THBS2-specific monoclonal antibodies. Chin. J. Immunol..

[22] Wang L., Zhang Y., Gao X., Duan Z., Wang S. (2010). Determination of Chloramphenicol Residues in Milk by Enzyme-Linked Immunosorbent Assay: Improvement by Biotin-Streptavidin-Amplified System. J. Agric. Food Chem..

[23] Oubiña A., Ballesteros B., Galve R., Barcelo D., Marco M.P. (1999). Development and optimization of an indirect enzyme-linked immunosorbent assay for 4-nitrophenol. Application to the analysis of certified water samples. Anal. Chim. Acta.

[24] Nemzek J.A., Siddiqui J., Remick D.G. (2001). Development and optimization of cytokine ELISAs using commercial antibody pairs. J. Immunol. Methods.

[25] Chen X., Xu L., Ma W., Liu L., Kuang H., Peng C., Wang L., Xu C. (2013). Development of an Enzyme-Linked Immunosorbent Assay for Cyhalothrin. Immunol. Investig..

[26] Jiang X.-X., Shi H.-Y., Wu N., Wang M.-H. (2011). Development of an enzyme-linked immunosorbent assay for diniconazole in agricultural samples. Food Chem..

[27] Rezapkin G., Dragunsky E., Chumakov K. (2005). Improved ELISA test for determination of potency of Inactivated Poliovirus Vaccine (IPV). Biologicals.

[28] Mukunzi D., Suryoprabowo S., Song S., Liu L., Kuang H. (2018). Development of an indirect enzyme-linked immunosorbent assay and lateral-flow test strips for pefloxacin and its analogues in chicken muscle samples. Food Agric. Immunol..

[29] Yao J., Sun Y., Li Q., Wang F., Teng M., Yang Y., Deng R., Hu X. (2017). Colloidal gold-McAb probe-based rapid immunoassay strip for simultaneous detection of fumonisins in maize. J. Sci. Food Agric..

[30] Wu W.-d., Li M., Chen M., Li L.-p., Wang R., Chen H.-l., Chen F.-Y., Mi Q., Liang W.-w., Chen H.-z. (2017). Development of a colloidal gold immunochromatographic strip for rapid detection of *Streptococcus agalactiae* in tilapia. Biosens. Bioelectron..

[31] Xing C., Jing X., Zhang X., Yuan J. (2017). Ultrasensitive indirect competitive ELISA and strip sensor for detection of furazolidone metabolite in animal tissues. Food Agric. Immunol..

[32] Liu L., Kuang H., Peng C., Wang L., Xu C. (2014). Structure-specific hapten design for the screening of highly sensitive and specific monoclonal antibody to salbutamol. Anal. Methods.

[33] Kim M.J., Lee H.S., Chung D.H., Lee Y.T. (2003). Synthesis of haptens of organophosphorus pesticides and development of enzyme-linked immunosorbent assays for parathion-methyl. Anal. Chim. Acta.

[34] Guo J., Liu L., Xue F., Xing C., Song S., Kuang H., Xu C. (2015). Development of a monoclonal antibody-based immunochromatographic strip for cephalexin. Food Agric. Immunol..

[35] Zhao C., Liu W., Ling H., Lu S., Zhang Y., Liu J., Xi R. (2007). Preparation of anti-gatifloxacin antibody and development of an indirect competitive enzyme-linked immunosorbent assay for the detection of gatifloxacin residue in milk. J. Agric. Food Chem..

[36] Wang Z.-X. (2019). Rapid Immunoassay of Nine Veterinary Drug Residues in Food. Ph.D Thesis.

[37] Fu Y.-J., Liu Z.-G., Wu Y.-X. (2010). Rapid detection of aureomycin residues in food by colloidal gold immunochromatography. Food Sci..

[38] Song S., Liu N., Zhao Z., Ediage E.N., Wu S., Sun C., De Saeger S., Wu A. (2014). Multiplex Lateral Flow Immunoassay for Mycotoxin Determination. Anal. Chem..

[39] Zhu Y., Li L., Wang Z., Chen Y., Zhao Z., Zhu L., Wu X., Wan Y., He F., Shen J. (2008). Development of an immunochromatography strip for the rapid detection of 12 fluoroquinolones in chicken muscle and liver. J. Agric. Food Chem..

